# Analysis of the assessment of antimicrobial susceptibility. Non-typhoid Salmonella in meat and meat products as model (systematic review)

**DOI:** 10.1186/s12866-021-02268-1

**Published:** 2021-08-02

**Authors:** Sandra M. Rincón-Gamboa, Raúl A. Poutou-Piñales, Ana K. Carrascal-Camacho

**Affiliations:** 1 Laboratorio de Microbiología de Alimentos. Grupo de Biotecnología Ambiental e Industrial (GBAI), Departamento de Microbiología, Facultad de Ciencias, Pontificia Universidad Javeriana, Bogotá D.C., Colombia; 2 Laboratorio Biotecnología Molecular. Grupo de Biotecnología Ambiental e Industrial (GBAI), Departamento de Microbiología, Facultad de Ciencias, Pontificia Universidad Javeriana, Bogotá D.C., Colombia

**Keywords:** Multidrug resistance, Meat products, Standard, Non-typhoidal Salmonella

## Abstract

**Background:**

The scientific publications of antimicrobial susceptibilities and resistance must be precise, with interpretations adjusted to the standard. In this frame, knowledge of antimicrobial resistance is fundamental in pathogenic microorganisms such as *Salmonella* spp., known for many annual deaths worldwide. The objective of this work was to compare the interpretation of standards, the concentrations, and the breakpoints, to study antimicrobial resistance in Non-Typhoidal Salmonella (NTS) isolated from beef, pork, and chicken meat, meat products, and propose additional considerations that improve the use and usefulness of published results.

**Results:**

After refining the search based on meeting the inclusion and exclusion criteria, 48 papers were selected. In 33 (68.8%) of them, the disc diffusion method was used, in 11 (22.9%) the MIC determination method, and in 4 (8.33%) were used both. In 24 (50%) of the articles, the selection of a different (correct) standard could have had an impact on the interpretation of antimicrobial susceptibility, which observed when considering three scenarios, i) comparison between the year of the isolation versus the implemented standard, ii) comparison between the year of submission versus implemented standard and iii) comparison between the year of publication versus implemented standard.

**Conclusions:**

The most frequent scenario was the inadequate selection of standards, indicating that some studies had not ensured that applied standards kept in line with the date of isolation, date of publication and interpretation of susceptibilities. We proposed 2 years for standards use for resistance and multi-resistance interpretations. On the other hand, we invite researchers to publish their results in the shortest possible time, and editors and reviewers of scientific journals to prioritise these types of studies and verify the correspondence between the standard cited and the one used and the one to be taken into account.

**Supplementary Information:**

The online version contains supplementary material available at 10.1186/s12866-021-02268-1.

## Abbreviations for antimicrobial agent reported in the different selected articles and standardized abbreviation and color used for each antimicrobial class in this work

**Table:** 

Antimicrobial agent	Abbreviations used in the papers	Abbreviations for this paper
**Penicillins**		
Ampicillin	AM, AMP, A, Amp, Ap	AMP
Ticarcilin	TIC, TC, TI, Ti	TIC
**β-Lactam/β-Lactamase inhibitor combinations**		
Amoxicillin/clavulanate	AUG, AMC, Amc, AC	AMC
Ampicillin-sulbactam	SAM, AS	SAM
Piperacillin-tazobactam	PPC-TAZ, TZP	TZP
**Cephems**		
Cefazolin	CFZ, KZ, CZ, CF, CZD	CFZ
Cephalothin	CF, CEP, CEF, KF	CEP
Cefepime	CPM, FEP	FEP
Cefotaxime	CTX, TAX, CT	CTX
Ceftriaxone	AXO, CRO, Co, CTR	CRO
Cefoxitin	FOX	FOX
Cefuroxime	FUR, CXM	CXM
Ceftazidime	CAZ, CTZ, CF	CAZ
Cefoperazone	CFP	CFP
Cefaclor	CEC, CFC	CFC
Cefpodoxime	CPD	CPD
**Monobactams**		
Aztreonam	ATM, AZT, AM	ATM
Ertapenem	ETP	ETP
Imipenem	IPM, IMP, IMI	IMI
Meropenem	MEM	MEM
**Aminoglycosides**		
Gentamicin	GM, G, CN, GE, Gm, GN	GEN
Tobramycin	TOB, To	TOB
Amikacin	AMI, AM, AMK, AN, Ak	AMK
Kanamycin	KAN, K	KAN
Streptomycin	S, STR, SM, EST	STR
**Fluoroquinolones**		
Ciprofloxacin	CIP, Cp, CI, CPF, CPX	CIP
Levofloxacin	Lvx	Lvx
Ofloxacin	OFX	OFX
Norfloxacin	NOR	NOR
**Quinolones**		
Nalidixic acid	NA, NAL, Nx, N	NAL
**Folate pathway inhibitors**		
Trimethoprim-sulfamethoxazole	SXT, COT, ST, TMP – SLF, TS	SXT
Sulfonamides	SSS, SMX, sul, SUL, SMX	SUL
Trimethoprim	TMP, TRIM, TP, W	TMP
**Phenicols**		
Chloramphenicol	C, CHL, CM, CLF, CLO, CRO	CHL
**Nitrofurans**		
Nitrofurantoin	FT, NIT	NIT
**Tetracyclines**		
Tetracycline	TE, TET, T, TCY	TET

## Background

Antimicrobial resistance monitoring programmes assess isolation of interest associated with foodborne diseases (FBD) against a range of antibiotics of importance in humans [1] to understand the dynamics of microorganisms in a community.

Among the national surveillance systems are the Canadian Integrated Program for Antimicrobial Resistance Surveillance (CIPARS), the National Antimicrobial Resistance Monitoring System (NARMS) since 1996 in the United States of America [2], and in Japan since 1999, the Japanese Veterinary Antimicrobial Resistance Monitoring System (JVARM) [3]. International systems include the European Antimicrobial Resistance Surveillance Network (EARSS, at present EARS-Net) funded in 1998 [4], and Latin America, the Latin American Antimicrobial Resistance Surveillance Network (ReLAVRA) since 1996 [5].

The known and approved methods for the assessment of antimicrobial susceptibility are the determination of the minimum inhibitory concentration (MIC) by dilution (broth micro- or macro dilution, or agar-dilution), the disc diffusion method (Kirby Bauer), and the MIC determination by epsilometric test (E-test) [6]. The first two methods recognised by CLSI (Clinical and Laboratory Standards Institute) and EUCAST (European Committee on Antimicrobial Susceptibility Testing) which are frequently updated the breakpoints and allow viewing and downloading of the latest editions [7].

At present, for epidemiological analysis of antimicrobial susceptibility tests, two types of criteria exist. The first the Food and Drug Administration (FDA) Epidemiological Cut-off Values (ECV). In this test, “the MIC value or zone diameter value separates microbial populations into those with and without acquired and or mutational resistance, based on their phenotypes (wild-type [WT] or non-wild-type [NWT]); the ECV defines the upper limit of susceptibility for the wild-type population of isolates” [8]. Second, The EUCAST Epidemiological Cut-off Values (ECOFF). In this test, “values separate the naive, susceptible wild-type bacterial populations from isolates that have developed reduced susceptibility to a given antimicrobial agent. The ECOFFs may differ from breakpoints used for clinical purposes, which are set out against a background of clinically relevant data, including therapeutic indication, clinical response data, dosing schedules, pharmacokinetics and pharmacodynamics” [9].

However, breakpoints, also known as clinical breakpoints, are specific parameter values, such as MIC or zone diameter value used, according to which isolation is clinically classified (interpretative criteria), such as “susceptible (S)”, “intermediate (I)” and “resistant (R)” [8, 10].

*Salmonella* spp. is recognised as one of the most important pathogens causing FBD [11]. Non-typhoidal Salmonella serovars (NTS) cause gastroenteritis in various hosts [12], including human bacteraemia [13]. One of the transmission routes of NTS is the consumption of contaminated food [7, 11, 14, 15] as fresh meat and eggs [14]. The main transmission route involves the evisceration and intestinal contents removal, where occurs cross-contamination [16–19]. This scenario becomes more complicated due to microorganism biofilms formation [14]. Of course, much care is necessary for the remaining stages of production: processing, distribution, sale and handling, to avoid the spread of the microorganism [18].

One big problem with pathogens causing FBD is antimicrobial resistance. In 2019, EFSA reported 87,923 cases of salmonellosis as the most frequent cause of FBD. The entity reported that 25.4% of the human isolates were MDR (multidrug-resistant) [9]. In addition, risk estimation data for 2019 in NARMS “Antibiotic Resistance Threats 2019” describes 212,500 annual cases of antibiotic-resistant non-typhoidal Salmonella infections and 70 deaths annually [20].

Among the factors that have influenced the emergence of antimicrobial resistance is the indiscriminate use of human antibiotics in livestock farming. It has been estimated in some countries that almost 50% of the antimicrobials produced worldwide are for livestock activities as prophylactics, growth promoters and in the treatment of diseases [18, 21, 22]. For example, Australia import about 700 tons each year and about 500 tons (78%) are used [22] in livestock activities. All this justifies the importance of monitoring and controlling the use of antibiotics.

In the USA, the Food and Drug Administration (FDA) and the Center for Veterinary Medicine (CVM) control the use of antimicrobials. However, the USA has approved 17 antimicrobials classes as growth promoters for animals. It includes tetracyclines, macrolides and penicillins [22].

In the light of the above, the present review intended to analyze and compare the interpretation of standards, the concentrations of antimicrobials used in each study, and the breakpoints, to study antimicrobial resistance in Non-Typhoidal Salmonella (NTS) isolated from beef, pork, and chicken meat and meat products, and to improve by proposals the use and usefulness of published results.

## Materials and methods

### Search strategy

The article searches were conducted in Web of Science (WoS), SCOPUS, Science Direct, and JSTOR. Regional documents searched in “*Biblioteca Virtual de Salud* (BVS)” and PubMed. Search equations used were based on the interaction of three groups: The first included Salmonella / zoonotic Salmonella / foodborne pathogen/ Salmonella; the second antimicrobial resistance, antibiotic resistance/ multidrug resistance, and the third meat products/ meat poultry/ pork/ beef, employing the Boolean operator “AND”.

For BVS and PubMed, the following browsers were used Descriptors in Health Sciences (DECS) and Medical subject headings (MESH), respectively, to perform searches for the terms as dependent variables: Salmonella food poisoning/“*intoxicación alimentaria por* Salmonella and *Salmonella enterica”.* Among independent variables associated with resistance drug resistance were employed microbial/“*farmacorresistencia microbiana”,* and Microbial sensitivity tests/“*pruebas de sensibilidad microbiana”*.

For the search associated with meat and meat products the terms used were: meat products*/“productos de carne”*, meat product*/“producto de carne”*, poultry products/“*productos avícolas”*, food safety/“*análisis de peligros y puntos de control críticos, inocuidad de los alimentos”*, food contamination/“*contaminación de alimentos”*, foodborne diseases/“*enfermedades transmitidas por los alimentos”*, fast foods/“*comidas rápidas”* and raw foods/“*alimentos crudos”*.

### Inclusion and exclusion criteria

Only included experimental studies performed between 1996 and 2019, covering the year that international observation and monitoring of antimicrobial resistance programs initiated. English and Spanish were the languages selected for articles, as described in the search equation. In the articles selected, the samples were meat or meat products collected at points of sale or intended for the same purpose and should identify *Salmonella* spp. and the non-typhoid serotype.

Selected articles were separated into two groups, the disc diffusion and minimal inhibitory concentration (MIC), to compare them. Likewise, was carrying out the verification of breakpoints and references for each of them (national or international). Articles that explicitly showed interpretative criteria or resistance frequency by isolate were selected.

Were excluded those articles whose title and abstract were unrelated to the present study. Articles involving isolations from collections were excluded due to lack of clarity on sample types, years of isolation or origin, such as food outbreak studies, as it was difficult to know the *Salmonella* spp., contamination source.

### Extraction and data registry

The information extracted was the following, the country of the study, the type of meat or meat product, the method used (disc diffusion or MIC determination), the breakpoints, the standard used and the regime applied (national or international) and antimicrobial susceptibility results.

Families of antibiotics considered belong to the following classes: Penicillin, β-lactam/β-lactamase inhibitor combinations, cephems, monobactams, aminoglycosides, quinolones, fluoroquinolones, folate pathway inhibitors, phenols, nitrofurans, and tetracyclines.

### Data analysis

Data analysis focused on three different facts: first, recognition of antimicrobial susceptibility test used, and national or international standard employed. Second, articles classified according to the method used disc diffusion or MIC determination for each group. Antibiotic concentration and breakpoints compared for each study. Additionally, the association between the date of submission, date of publication, and the year of the isolates versus the CLSI standard implemented. Third, was investigated the multi-resistance patterns and the percentage of prevalence according to serotype, taking into account the method described in each study.

Also suggested an optimal period of 2 years for the appropriate CLSI standard use, considering that the CLSI annually updates the M100 standards and includes a “tentative” period of 1 year for the manufacturer to implement the standard modifications. Additionally, we suggest that the peer review processes and the publication of antimicrobial resistance studies should not take more than 1 year, which would add up to the 2 years for the optimal period that we are proposing for standards use.

On the other hand, some studies have used the standard for the antimicrobial susceptibility evaluation of isolates from animal origin. M31 standard version was modified in 2013 and replaced by the VET01-S3 (2013). Subsequently, since June 2018, VET 01–04 (2018) changed into the VET08. Therefore, it is necessary to emphasize that to maintain the original information from the selected articles, in this work, the standard used was as it was published (M31) by authors.

## Results

### Number of articles, countries, and standard

A total of 3802 articles were related to the topic, 1141 (30%) were preselected, and only 4% (48/1141) met all the defined inclusion and exclusion criteria (Tables 1 and 2).

**Table 1:** Strategy for selection of eligible articles

Selection strategy	No. Articles (%)
1. All records identified through the search	3802
Articles duplicated in the searching process	2661/3802 (70%)
2. Number of preselected articles	1141/3802 (30%)
Inclusion and exclusion criteria	
The article title is not related to the topic of interest	857
The language of the article is different from English or Spanish	10
The article was not original	36
The publication of the article is out of the period 1996–2019	10
The origin of the sample is not clear	110
The technique used is not disk diffusion or MIC	3
The authors did not describe the breakpoints used in the article	13
The authors did not describe the correlation between serovar and antimicrobial resistance	54
3. Total of articles removed under the inclusion and exclusion criteria	1093/1141 (96%)
4. Total of articles selected	48/1141(4%)

**Table 2:** Standards used to define the interpretation criteria for antimicrobial susceptibility tests in selected articles

Article selected	Standards used			Antimicrobial susceptibility test		Country of study
	International programs	CLSI, M100	CLSI, M 31	Minimum Inhibitory Concentration	Disk Diffusion	
[23]		CLSI, M100-S11 [24]		X		Turkey
[25]	CASFM [26]				X	Senegal
[27]		CLSI, M100-S15 [28]			X	Spain
[29]		CLSI, M100-S15 [28]	CLSI, M31-A2 [30]		X	Vietnam
[31]		CLSI, M100-S15 [28]			X	Brazil
[32]	NARMS (it was not cited by authors); CIPARS [33]			X		Canada
[34]		CLSI, M100-S16 [35]; M100-S13 [36]			X	Iran
[37]		CLSI, M100-S19 [38]		X	X	Venezuela
[39]		CLSI, M100-S16 [35]			X	Thailand
[40]			CLSI, M31-A2 [30]	X		United States
[41]		CLSI, M100-S9 [42]			X	South Korea
[43]			CLSI, M31-S1 [30]	X		Brazil
[44]		CLSI, M100-S20 [45]		X		Canada
[46]		CLSI, M100-S20 [45]			X	Vietnam
[47]		CLSI, M100 - S21 [48]			X	South Korea
[49]			CLSI, M31-A3 [50]		X	Greece
[51]			CLSI, M31-A2 [30]		X	Spain
[52]		CLSI, M100-S18 [53]			X	Mexico
[54]	EUCAST [55]			X		Portugal
[56]		CLSI, M100 - S21 [48]			X	China
[57]		CLSI, M100-S16 [35]			X	China
[58]		CLSI, M100-S22 [59]			X	South Korea
[60]		CLSI, M100 - S21 [48]			X	Egypt
[61]		CLSI, M100-S17 [62]			X	Italy
[63]^a^		CLSI, M100-S23 [64]		X	X	Colombia
[17]		CLSI, M100-S22 [59]			X	Egypt
[65]		CLSI, M100 - S21 [48]			X	Vietnam
[66]	NARMS [67]		CLSI, M31-A3 [50]	X		China
[16]		CLSI, M100-S11 [24]			X	Egypt
[68]		CLSI, M100-S16 [35]			X	Iran
[69]		CLSI, M100-S23 [64]		X	X	
[70]		CLSI, M100-S22 [59]		X	X	Colombia
[71]		CLSI, M100-S20 [45]			X	Egypt
[72]			CLSI, M31-A3 [50]	X		Romania
[73]		CLSI, M100-S23 [64]			X	China
[74]		CLSI, M100 -S24 [75]		X		Egypt
[76]		CLSI, M100-S23 [64]			X	Thailand
[19]		CLSI, M100-S23 [64]			X	Vietnam
[77]			CLSI, M31-A3 [50]; CLSI, M31-S1 [78]		X	Malaysia
[79]		CLSI, M100-S25 [80]			X	Vietnam
[81]		CLSI, M100 - S21 [48]			X	Egypt
[82]		CLSI, M100 - S21 [48]		X		United States
[83]^b^		CLSI, M100-S23 [64]			X	China
[84]		CLSI, M100 -S24 [75]		X		Vietnam
[85]		CLSI, M100-S22 [59]			X	Malaysia
[86]		CLSI, M100 -S24 [75]			X	Singapore
[87]		CLSI, M100-S23 [64]			X	China
[88]		CLSI, M100-S28 [8]			X	China

^a^The authors refer to the standard “CLSI, M100-S2”, regarding the year of the appointment, the CLSI, M100-S23 is used for the analysis. ^b^The authors refer in the text to the CLSI standard, 2013; however, it does not appear in the bibliographic references, for this reason, it is assumed that the standard used following the citation is CLSI, M100-S23

When separating the articles according to the standard used to determine the susceptibility of the isolation, based on the breakpoints, we found that: 45/48 (93.8%) used the CLSI standards, 1/48 (2.08%) the NARMS or CIPARS standards, and 1/48 (2.08%) by *Comité de l’antibiogramme de la société française de microbiologie* (CA-SFM), and the European Committee on Antimicrobial Susceptibility Testing – EUCAST (Table 2).

Additionally, of the articles that used CLSI standards, 82.2% (37/45) used the M100, 7/45 (15.6%) used the M31 corresponding to disc diffusion and dilution susceptibility, and 1/45 (2.2%) used both standards [29] (Table 2).

### Analysis of antibiotic concentration used according to each method

#### Disc diffusion method

The disc diffusion method grouped the highest number of articles with 68.8% (33/48). The studies that implemented the disc diffusion method used the same concentration (30 μg) in Cephems (CFZ, CEP, FEP, CTX, CRO, FOX, CXM, and CFP), in Monobactams (ATM), in Quinolone (NAL), and Phenolic Compounds (CHL). While in Aminoglycosides (TOB 10 μg and AMK 30 μg) and Fluoroquinolones (OFX 5 μg). However, for each GEN, SXT, SUL, AMP, CAZ, IMI, STR, TMP, AMC, CFP, SAM, CIP, KAN, NOR, CPD, three different situations were observed:

1. In Zdragas et al. (2012), for CFP (cephems), the concentration cited was 30 μg, yet in the CLSI, M31-A3 (referred standard), the antibiotic is not included.
2. Among the revised articles, the most occurring non-conformance to the standards was a discrepancy between the concentration used in the study and the one referenced.
  - In Bada-Alambedji, et al. (2006), SXT (1.25–23.75 μg) concentration coincided between the Distributor (Bio-Rad) and the C.A.-S.F.M standard used. However, the antimicrobial concentration reported in the article was 1.25–25.75 μg.
  - In Dallal et al. (2010), AMP (10 μg), CAZ (30 μg), IMI (10 μg), STR (10 μg), TMP (5 μg), AMC (20/10 μg), TET (30 μg) concentrations were similar between the Distributor (Mast Diagnostics) and those presented in the CLSI M100-S16, 2006 and CLSI M 100-S13 standard. However, those reported in the article differed from CLSI M100-S16, 2006 and CLSI M 100-S13 standard for AMP (30 μg), CAZ (100 μg), IMI (30 μg), STR (15 μg), TMP (15 μg), AMC (75/10 μg), TET (15 μg).
  - In Molina et al., (2010), the concentration reported for AMC in the CLSI, M100-S19 standard and the distributor’s insert (BBL) was 20/10 μg. However, the antimicrobial concentration reported in the article was 75/10 μg.
  - Cabrera-Díaz et al., (2013), used a concentration of 30 μg for CIP. However, the BBL, BD, Sparks, MD companies and the CLSI M100-S18 standard specify the use of discs containing 5 μg CIP.
  - In Moawad et al., (2017), the concentration used for AMC and CPD was 20 μg, but that described by Oxoid and the CLSI, M100-S21 standard was of 20/10 μg and 10 μg, respectively.
  - In Zhang et al.*,* (2018), the concentration used for FEP was 5 μg, but the one described by the CLSI, M100:S23 standard references, and Oxoid (distributor) was 30 μg.
  - In Aihua Zhu et al., (2019), NOR concentration described in the article was 5 μg, but the disc content defined by the CLSI, M100: S23 standard and Oxoid (distributor) was 10 μg.
3. Discrepancies were between the disc content reported in the article and the one defined by the standard used. These articles not mentioned discs distributor.
  - In Kim et al. (2011), SAM is reported concentration was 30 μg, yet the one established by the cited standard (CLSI, M100-S21) was 10/10 μg.
  - In Li et al., (2014), for KAN and NOR, they reported 20 and 5 μg, respectively. However, those reported by the cited CLSI, M100-S21 standard were 30 and 10 μg, respectively.
  - In Cai et al., (2016), they reported a concentration of 5 μg for NOR, and that defined by the cited CLSI, M100-S23 standard was 10 μg.

#### MIC method

22.9% of the articles implemented MIC method (11/48), and 8.3% (4/48) used MIC and Disk Diffusion (Table 2). In articles to MIC determination, in 7/15 (46.6%) of them, the test range were not indicated [32, 37, 43, 69, 72, 74, 84]. Information regarding the remaining seven articles appears in Table S1.

#### Breakpoints and interpretative criteria for MIC

When comparing the breakpoints for *Salmonella* spp., by the two methodologies, using the CLSI standards, in general terms, we observed that there was a variation between 2010 and 2019 for Monobactams, Cephems, Fluoroquinolones, and Tetracyclines. These changes started from the M100-S20 standard on (Tables S2A and B). However, some situations became evident concerning MIC determination:

A. In the group of the articles using MIC determination, 4/11 (36.4%) [40, 43, 66, 72] implemented the CLSI, M31 standard. These works employed CTX, CRO, FOX, CAZ, ATM, STR, CIP, NAL, TMP, NIT that are not in the standard (Table S3). Therefore, the breakpoints used in the interpretation of susceptibility tests are unknown.
B. In the four articles (Table 2), where isolates were analyzed by MIC determination and disc diffusion methods and evaluate the antimicrobial resistance and β-lactamases production; the breakpoints for AMP, AMC, TZP, FEP, FOX, GEN, AMK, and SXT were like those described in CLSI, M100-S19, M100-S22, and M100-S23. In these standards, breakpoints for the antibiotics did not vary in 2009, 2012 and 2013 (Tables S2 A and B).

#### Comparison among isolation collection date, date of manuscript submission, date of publication, and implemented CLSI standard

To evaluate the association between the implemented CLSI M100 standard for 37/48 (77.1%) of the studies and their possible scenario, the standard used were associated with the date of isolation, date of manuscript submission, and publication date. We observed that:

#### First scenario: comparison between the year of the isolation and the standard applied

A comparison of 26/37 (70.3%) of the articles appears in Additional file 2. In 5/37 (13.5%) was not described the isolation date. In 6/37 (16.2%), the isolates were collected in different years and included in the same study.

The articles using the CLSI standard within the optimal considered period were 16/26 (61.5%). According to our proposed period, in 4/26 (15.4%) of the studies, the standards were wrong since they were 2–8 years outdated concerning the optimal period.

#### Second scenario: comparison between the year of submission of the article versus implemented standard

The analysis of the year of submission versus the used standard included only 29/37 articles (78.4%), as no description of date of submission appear for 8/37 (21.6%) articles.

In Additional file 2, 15/29 (51.7%) studies were among the optimal range. Of these, the study published by Molina et al., (2010), in our criteria, used the correct standard CLSI, M100-S19 (2009). However, from the following year onwards, breakpoints modifications appear in the CLSI standard, M100-S20 (2010), (Tables S3A and B), [31]; however, were not considered in the article.

#### Third scenario: comparison between the year of publication versus implemented standard

When the 37 articles using the M100 standard compared, we observed 13/37 (35%) of the studies state in range with the standards. In contrast, 24/37 (65%) of the studies did not use adequate ones concerning the year of publication. Yet, it is necessary to consider publishing processes are usually lengthy (Additional file 2).

#### Studies where the use of the suggested standards could have an impact

The total number of studies where the correct standard use could impact was 24 (50%). Concerning this, different scenarios appear below.

First scenario. According to our criteria, in 4/26 (15.4%) publications, the standard used was inadequate since the implemented standard was 2–8 years outdated concerning the optimal period. On the other hand, 6/26 (23.1%) of the articles use a standard closer to the submission date rather than the isolation date. As a case in point, Gad et al., (2018) obtained their isolated in 2009, the CLSI standard used was M100-S21 (2011) and the manuscript submitted in 2018 (Table 3).

**Table 3:**
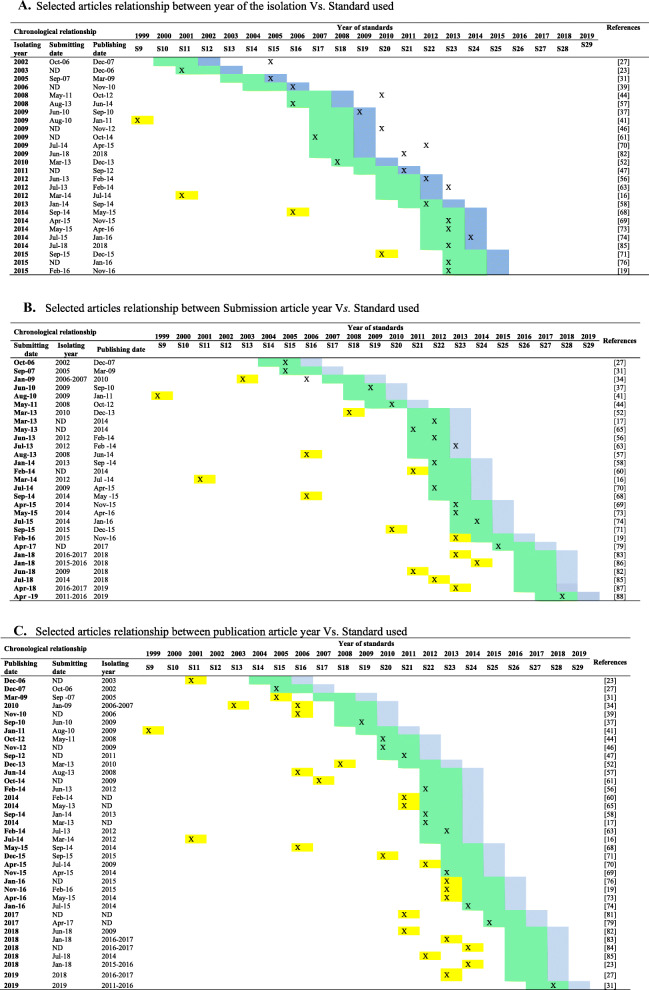
Articles that used a different standard than the one suggested and its impact on the interpretation of susceptibility tests

^a^ The data of the breakpoints analyzed were those described by the authors. *S* susceptible, *I* intermediate, *R* resistant

Second scenario. According to our criteria, 14/29 articles (48.3%) implemented an inadequate standard, as they were outside the range considered optimal. Moreover, in 13 studies, the interpretation of the results could change according to the breakpoints used (Table 3).

Third scenario. According to our criteria, 24/37 (65%) of the studies did not use the adequate standard concerning the year of publication. Yet it is necessary to take into account publishing processes are usually lengthy (Table 3).

Table 3 contains the 24 references, where using a different standard could have had an impact. Besides, we specify 56 cases where the interpretation may have been other than the published one. In 2/24 (8.3%) of studies, utilization of the suggested standard would not have caused a change in the interpretation of antimicrobial susceptibility because antibiotics used did not change (comparing the standard applied and the suggested one) (Table 3).

In 10/24 studies (41.6%), when analysing the antibiotics described and comparing them with the suggested norms within the optimal period, it was observed that there were no breakpoint values for STR, OFX, Lvx, NOR, CEP, and NAL (Table 3). By 2006 in M100-S16 says for STR that “aminoglycosides may appear active in vitro, but are not clinically effective and should not be reported, as susceptible”. M100-S23 says that re-evaluation of fluoroquinolones OFX and Lvx was ongoing. In M100-S26, antimicrobials such as NOR, CEP, and M100-S27, antimicrobials as NAL ceased to assayed for *Salmonella* spp.

During the study, we found 56 cases of antibiotics with possible changes in the susceptibility interpretation. In 2 (3.6%) cases, there was no impact. In other 2 (3.6%) cases, the suggested standard had different values in Moawad et al., (2017) for AMP, AMC, and SXT or lower/ higher values in Fakhr et al., (2006) for GEN, KAN, SXT, and SUL. In 17 (30.3%) cases, breakpoints were no found in the suggested standard. In 8 (14. 3%) cases, the interpretation could have changed from I to S or from R to I by applying the suggested standard. For 27 (48.2%) cases, the interpretive criteria could have changed from S to I or from I to R, once implemented the appropriate standards (Table 3).

## Discussion

### Antibiotic concentration analysis according to each method used

The most frequently used method in the articles was disc diffusion. However, MIC determination is the most recommended in monitoring programmes because quantitative tests are more accurate [89, 90], although disc diffusion tests are easier to perform and cheaper [89, 90].

Upon comparison, in both methods (Table 2), the antimicrobial agent concentration varied depending on the system, the distributor, and the methodology (as expected). 8.3% of the remaining articles used both techniques in a combined manner to complement antimicrobial susceptibility tests and to detect ß-lactamases in food, a logic strategy in many studies. However, in some studies [25, 34, 37, 52, 73, 81, 83, 87, 91], used antibiotic concentration in disc diffusion assays were different than the described in the standard referenced by those authors, suggesting the antimicrobial susceptibility interpretation (resistant, intermediate, or susceptible) lacks or lost the support of the breakpoints described in the standard.

### Comparison of breakpoints and interpretative criteria for antimicrobial susceptibility tests

In 2017 the WHO, FAO, and the World Organization for Animal Health designed a guide for integrated surveillance of antimicrobial resistance in pathogenic bacteria responsible for foodborne diseases. This guideline establishes the antimicrobial surveillance perform of the clinical samples collected in livestock production from animal and environmental samples and in the finished product (food of animal origin) distributed in the retail trade (this being the most frequent route of human contamination). Also, the guideline mentions efforts to harmonise the interpretive criteria for antimicrobial susceptibility testing to get comparable data [92].

Usage of standards for both MIC-determination and disc diffusion methods and the inter and intra-laboratory quality control systems should lead to reproducible results [93]. Hence, the importance of complying with test specifications, quality control management, and updated standard version.

It is crucial to know that CLSI established breakpoints depending on the behaviour of pathogens worldwide, varying as a function of study and analysis by an international committee of experts. CLSI M100 changed breakpoints and interpretative criteria for Penicillins, Cephems, Monobactams, Fluoroquinolones, and Tetracyclines between 2006 and 2019 (Tables S3 A and B). The crucial change observed from the CLSI M100-S20 onwards was for the Cephems. This information is critical because an inadequate standard use; could result in over or underestimation of identified resistances.

On the other hand, in the articles [40, 43, 66, 72], the CLSI standard M31 was referred for defining the breakpoints of CTX, CRO, FOX, CAZ, ATM, STR, CIP, NAL, TMP, NIT; which are not in M31.

### Comparison between isolate collection, article submission, and publication with the implemented CLSI standard

In the development of antimicrobial susceptibility tests, the laboratory is responsible for using the current CLSI standard or the standard to be implemented, and rigorously follow the system’s instructions by the manufacturer, as well as a strict adhesion to the established procedure to accordingly classify the isolate as susceptible, intermediate or resistant [94].

Table 3 shows 24 studies where the standard used did not coincide with the suggested and microbial susceptibility interpretation. From these, 8.3% (2/24) did not impact the results since the antimicrobials employed did not have breakpoints modifications for the proposed standard versus the implemented one. 11/24 (45.8%) of the impact of the articles was on the susceptibility interpretation to cephems. 7/24 (29.2%) articles impact was on the tetracyclines susceptibility interpretation. In 2/24 (8.3%) articles involve the susceptibility interpretation to folate pathway inhibitors. 1/24 (4.2%) articles impacted on the susceptibility interpretation to aminoglycosides.

Additionally, in the article by Yu et al., (2014) if using of suggested M100-S21, −S22, −S23, −S24 standards, “intermediate” isolate interpretation for ATM could change to “susceptible”. However, the study does not report intermediate isolates. On the other hand, in the article by Sodagari et al., (2015), all isolates were reported as susceptible to IMI; yet this interpretation can change as resistant if any of the M100-S22, −S23, −S24 standard would have implemented, as suggested in the optimal period proposed.

Studies by Cabrera-Diaz et al., (2013), Abd-Elghany et al., (2015), Yu et al., (2014), Sodagari et al., (2015), Gharieb et al., (2015) used CIP within the antimicrobial susceptibility tests. When analysed the susceptibility of isolates to CIP by using M100-S18, −S11, −S16, −S15 and -S20 standards (used by the authors) and M100-S22, −S23, −S24 and -S25 (suggested standards); the interpretation changed from susceptible to intermediate and from intermediate to resistant.

According to Jorgensen & Turnidge (2015), the clinic isolates classified as “intermediate” can be inhibited by non-toxic attainable antimicrobial concentration if the dosage is high or administration prolonged. Antimicrobials also can be safely used when the infection is in a site where the medication can remain physiologically concentrated (for example, the urinary tract).

Additionally, antimicrobials with “intermediate” results also can be used as a buffer zone in the interpretation, avoiding minor mistakes in technical factors causing discrepancies in antimicrobial susceptibility interpretation. For example, from “susceptible to intermediate” instead of from “susceptible to resistant” [92, 95], where the modification from “susceptible” to “intermediate” would not have an impact, as other interpretative mistakes would.

In contrast, when the isolate is mistakenly reported as “susceptible” when it is “resistant”, it is a big problem. In humans, *S*. Typhimurium isolates resistant to antimicrobials have been associated with an increased risk of infection, frequent hospitalization, disease, and risk of death, in contrast to susceptible *S*. Typhimurium isolates. Therefore, accurate, rapid, cost-effective classification of multi-resistant isolates is necessary for illness management [96].

The finding of food antimicrobial-resistant isolates, especially from livestock productions and production environments, have a human health impact due to the responsibility for the failure of human treatments and the capacity to generate disease. Additionally, in some studies, authors have described the spread of resistance genes through isolates obtained from food and environments to the intestinal microbiota [97, 98]. Hence, the importance of correct classification in antimicrobial susceptibility test as a source of information for antimicrobial resistance surveillance programs. However, in certain versions of CLSI standards supplements, the underlying method may change. Hence, the need for prior verification before using an updated version of the standards.

Antimicrobial resistance may be natural and evolutionary; however, the imprudent use of antimicrobials has accelerated it. In this regard, some authors predicted that by 2050 there could be close to 10 million deaths due to infections caused by antimicrobial-resistant microorganisms, a clinical situation that could be worst because of COVID 19 [99, 100].

Therefore, studies assessing antimicrobial resistance must be rigorous and describe aspects such as those highlighted by Van et al., (2007): sampling procedures, sample type, identification methods and selection of the appropriate standard, among others, as described in this review.

## Conclusions

According to our criteria, the inappropriate standard selection was the most frequent scenario (date of publication vs standard implemented). We remark the necessity to review the standards employing, to assure they are in line with the isolation and publication dates, demonstrating whether there could be interpretations changes.

This systematic review proposed an optimal period of 2 years in the standard to use for multi-resistance interpretation to be homogenous since multiresistant isolates are the most virulent. Thus, they are known as “superbugs” [101]. In this regard, we call on researchers to publish their antimicrobial susceptibility results in the shortest time possible. For longitudinal retrospective studies, it is crucial to use the correct and current standard, according to isolation date, making the necessary clarifications.

We also suggest to editors and scientific journal reviewers to prioritize these types of studies. Besides, verify the correspondence between the cited standard and the one that should employ, knowing that international standards are usually updated every year. This systematic review also suggests that other publications regarding antimicrobial resistance of some other pathogenic microorganisms could be presenting the same discrepancies we describe.

Finally, we propose the generation of an international, codified and easily accessible database where researchers can record the results of antimicrobial susceptibility testing of isolates from different links in the production chain. This database should include the date and origin of the isolates, their identification, the methodology used and the standard used. It would also be interesting if the database could automatically process the results. It is clear that the database proposal is ambitious, but it would allow for better collaborative work on antimicrobial resistance trends.

## Supplementary Information

**Additional file 1.**

**Additional file 2:.** A. Selected articles relationship between year of the isolation Vs. Standard used. B. Selected articles relationship between Submission article year Vs. Standard used. C. Selected articles relationship between publication article year Vs. Standard used [16, 17, 19, 23, 27, 31, 34, 37, 39, 41, 44, 46, 47, 52, 56–58, 60, 61, 63, 65, 68–71, 73, 74, 76, 79, 81–88]

## Data Availability

The results and data are mostly presented in the document and in the supplementary material; however, they are available upon request.
