# Supplementary material for: Analysis of the assessment of antimicrobial susceptibility. Non-typhoid Salmonella in meat and meat products as model (systematic review)
Source: BMC Microbiol. 2021 Aug 2;21:223. doi: 10.1186/s12866-021-02268-1 (PMC8328484; doi:10.1186/s12866-021-02268-1)
Supplement: Supplementary file 1 — Additional file 1. [file 12866_2021_2268_MOESM1_ESM.docx]

**Supplementary material**

**Analysis of the assessment of antimicrobial susceptibility. Non-Typhoid *Salmonella* in meat and meat products as an example (Systematic review)**

Sandra M. Rincón-Gamboa^1,2^, Raúl A. Poutou-Piñales^2^*, Ana K Carrascal-Camacho^1^

^1^ Laboratorio de Microbiología de Alimentos. Grupo de Biotecnología Ambiental e Industrial (GBAI). Departamento de Microbiología, Facultad de Ciencias, Pontificia Universidad Javeriana. Bogotá D.C., Colombia.

^2^ Laboratorio Biotecnología Molecular. Grupo de Biotecnología Ambiental e Industrial (GBAI). Departamento de Microbiología, Facultad de Ciencias, Pontificia Universidad Javeriana. Bogotá D.C., Colombia.

*Corresponding Author

Raúl A. Poutou-Piñales, Ph.D.

Professor

*E-mail: [rpoutou@javeriana.edu.co](mailto:rpoutou@javeriana.edu.co)

Pontificia Universidad Javeriana, Bogotá, D.C., Colombia

Facultad de Ciencias

Departamento de Microbiología. Grupo de Biotecnología Ambiental e Industrial (GBAI).

Laboratorio de Biotecnología Molecular

Carrera 7ma No 43-82, Edificio 50 Lab. 124

Código Postal: Bogotá 110-23

Fax: 57-1 320 83 20 ext: 4021

Table S1.

The concentration of antimicrobial agent used in the antimicrobial susceptibility test by MIC

| **Antimicrobial agent** | **Automated methods** | | | | **CLSI reference method** | |
| --- | --- | --- | --- | --- | --- | --- |
|  | **Trek diagnostics, Westlake, OH**  **μg/mL** | **Reference** | **Phoenix NMIC/ID-121****  **μg/mL** | **Reference** | **Agar dilution method**  **μg/mL** | **Reference** |
| AMP | 1-32 | [1-5] | 4-16 | [6, 7] | 0.5-64 | [8] |
|  |  |  |  |  | 4-32 | [9] |
| AMC | 1-32/0.5-16 | [2-5] | 4/2-16/8 |  | 4/2-32/16 |  |
| CEP | 2-32 | [3, 5] | 1-16 |  |  |  |
| CTX |  |  | 4-32 |  | 0.06-8 | [8] |
|  |  |  |  |  | 4-64 | [9] |
| CRO | 0.25-64 | [1-5] | 2-32 |  | 2-64 |  |
| FOX | 0.5-16 | [3, 5] | 4-16 |  | 4-32 |  |
|  | 0.5-32 | [1, 2, 4] |  |  |  |  |
| GEN | 0.25-16 | [1-5] | 2-8 |  | 0.25-0.32 | [8] |
|  |  |  |  |  | 2-16 | [9] |
| AMK | 0.5-4 | [3, 5] | 8-32 |  | 8-64 |  |
|  | 0.5-64 | [1] |  |  |  |  |
| KAN | 8-64 | [1-5] |  |  | 4-64 |  |
| STR | 32-64 |  |  |  | 32-64 |  |
|  |  |  |  |  | 2-512 | [8] |
| CIP | 0.015-4 | [2, 3] | 0.5-2 |  | 0.008-8 |  |
|  | 0.5-4 |  |  |  | 1-8 | [9] |
| NAL | 0.5-32 |  |  |  | 2-512 | [8] |
|  |  |  |  |  | 4-32 | [9] |
| SXT | 0.12/4-2.38/76 |  | 0.5/9.5-2/38 |  | 0.5/9.5-4/76 |  |
| SUL | 16-512 | [3, 5] |  |  | 64-512 |  |
| TMP |  |  |  |  | 0.25-32 | [8] |
| CHL | 2-32 | [1, 3-5] |  |  | 2-256 |  |
|  |  |  |  |  | 4-32 | [9] |
| TET | 4-32 |  | 2-8 |  | 0.5-64 | [8] |
|  |  |  |  |  | 2-16 | [9] |

Table S2.

1. Variation in breakpoints in the CLSI standards by MIC

| **Year of publication of the Standard** | **Standard**  **CLSI M100** | **Penicillins**  **μg/mL** | | | **Cephems**  **μg/mL** | | | | | | | | | | | | | | | | | | **Monobactams**  **μg/mL** | | | | | | | | | | | | **Fluoroquinolones**  **μg/mL** | | | | | | | | | | | |
| --- | --- | --- | --- | --- | --- | --- | --- | --- | --- | --- | --- | --- | --- | --- | --- | --- | --- | --- | --- | --- | --- | --- | --- | --- | --- | --- | --- | --- | --- | --- | --- | --- | --- | --- | --- | --- | --- | --- | --- | --- | --- | --- | --- | --- | --- | --- |
|  |  | **TIC** | | | **CFZ** | | | **CEP** | | | **FEP** | | | **CTX** | | | **CRO** | | | **CAZ** | | | **ATM** | | | **ETP** | | | **IMI** | | | **MEM** | | | **CIP** | | | **Lvx** | | | **OFX** | | | **NOR** | | |
|  |  | S | I | R | S | I | R | S | I | R | S | I | R | S | I | R | S | I | R | S | I | R | S | I | R | S | I | R | S | I | R | S | I | R | S | I | R | S | I | R | S | I | R | S | I | R |
| 2006-2008 | S16 - S18 | ≤ 16 |  | ≥ 128 | ≤ 8 |  | ≥ 32 | ≤ 8 |  | > 32 | ≤ 8 |  | > 32 | ≤ 8 |  | ≥ 64 | ≤8 |  | ≥64 | ≤ 8 |  | ≥ 32 | ≤ 8 |  | ≥ 32 | ≤ 2 |  | ≥ 8 | ≤ 4 |  | ≥ 16 | ≤ 4 |  | > 16 | ≤ 1 |  | ≥ 4 | ≤ 2 |  | ≥ 8 | ≤ 2 |  | ≥ 8 | ≤ 4 |  | > 16 |
| 2010 | S20 | ≤ 16 | 32-64 | ≥ 128 | ≤ 1 | 2 | ≥ 4 | ≤ 8 | 16 | > 32 | ≤ 8 | 16 | > 32 | ≤ 1 | 2 | ≥ 4 | ≤ 1 | 2 | ≥ 4 | ≤ 4 | 8 | ≥ 16 | ≤ 4 | 8 | ≥ 16 | ≤ 2 | 4 | ≥ 8 | ≤ 4 | 8 | ≥ 16 | ≤ 4 | 8 | > 16 | ≤ 1 | 2 | ≥ 4 | ≤ 2 | 4 | ≥ 8 | ≤ 2 | 4 | ≥ 8 | ≤ 4 | 8 | > 16 |
| 2011 | S21 | ≤ 16 | 32-64 | ≥ 128 | ≤ 2 | 4 | ≥ 8 | ≤ 8 | 16 | > 32 | ≤ 8 | 16 | > 32 | ≤ 1 | 2 | ≥ 4 | ≤ 1 | 2 | ≥ 4 | ≤ 4 | 8 | ≥ 16 | ≤ 4 | 8 | ≥ 16 | ≤ 0.25 | 0.5 | ≥ 1 | ≤ 1 | 2 | ≥ 4 | ≤ 1 | 2 | ≥ 4 | ≤ 1 | 2 | ≥ 4 | ≤ 2 | 4 | ≥ 8 | ≤ 2 | 4 | ≥ 8 | ≤ 4 | 8 | > 16 |
| 2012 | S22 |  |  |  | ≤ 2 | 4 | ≥ 8 | ≤ 8 | 16 | > 32 | ≤ 8 | 16 | > 32 | ≤ 1 | 2 | ≥ 4 | ≤ 1 | 2 | ≥ 4 | ≤ 4 | 8 | ≥ 16 | ≤ 4 | 8 | ≥ 16 | ≤ 0.5 | 1 | >2 | ≤ 1 | 2 | ≥ 4 | ≤ 1 | 2 | ≥ 4 | ≤ 0.06 | 0.12-0.5 | ≥ 1 |  |  |  | ≤ 2 | 4 | ≥ 8 | ≤ 4 | 8 | > 16 |
| 2013 | S23 | ≤ 16 | 32-64 | ≥ 128 | ≤ 2 | 4 | ≥ 8 | ≤ 8 | 16 | > 32 | ≤ 8 | 16 | > 32 | ≤ 1 | 2 | ≥ 4 | ≤ 1 | 2 | ≥ 4 | ≤ 4 | 8 | ≥ 16 | ≤ 4 | 8 | ≥ 16 | ≤ 0.5 | 1 | >2 | ≤ 1 | 2 | ≥ 4 | ≤ 1 | 2 | ≥ 4 | ≤ 0.06 | 0.12-0.5 | ≥ 1 | ≤ 0.12 | 0.25 -1 | ≥ 2 | ≤ 0.12 | 0.25 -1 | ≥ 2 | ≤ 4 | 8 | > 16 |
| 2014 -2015 | S24 - S25 | ≤ 16 | 32-64 | ≥ 128 | ≤ 2 | 4 | ≥ 8 | ≤ 8 | 16 | > 32 | ≤ 2 |  | > 16 | ≤ 1 | 2 | ≥ 4 | ≤ 1 | 2 | ≥ 4 | ≤ 4 | 8 | ≥ 16 | ≤ 4 | 8 | ≥ 16 | ≤ 0.5 | 1 | >2 | ≤ 1 | 2 | ≥ 4 | ≤ 1 | 2 | ≥ 4 | ≤ 0.06 | 0.12-0.5 | ≥ 1 | ≤ 0.12 | 0.25 -1 | ≥ 2 | ≤ 0.12 | 0.25 -1 | ≥ 2 | ≤ 4 | 8 | > 16 |
| 2016 -2019 | S26 - S29 |  |  |  | ≤ 2 | 4 | ≥ 8 |  |  |  | ≤ 2 |  | > 16 | ≤ 1 | 2 | ≥ 4 | ≤ 1 | 2 | ≥ 4 | ≤ 4 | 8 | ≥ 16 | ≤ 4 | 8 | ≥ 16 | ≤ 0.5 | 1 | >2 | ≤ 1 | 2 | ≥ 4 | ≤ 1 | 2 | ≥ 4 | ≤ 0.06 | 0.12-0.5 | ≥ 1 | ≤ 0.12 | 0.25 -1 | ≥ 2 | ≤ 0.12 | 0.25 -1 | ≥ 2 |  |  |  |
|  |  |  |  |  |  |  |  |  |  |  |  |  |  |  |  |  |  |  |  |  |  |  |  |  |  |  |  |  |  |  |  |  |  |  |  |  |  |  |  |  |  |  |  |  |  |  |

Green boxes highlight differences between standards

1. Variation in breakpoints in the CLSI standards by Disk Diffusion

| **Year of publication of the Standard** | **Standard**  **CLSI M100** | **Penicillins**  **mm** | | | **Cephems**  **mm** | | | | | | | | | | | | | | | | | | **Monobactams**  **mm** | | | | | | | | | | | | **Fluoroquinolones**  **mm** | | | | | | | | | | | | **Tetracyclines**  **mm** | | |
| --- | --- | --- | --- | --- | --- | --- | --- | --- | --- | --- | --- | --- | --- | --- | --- | --- | --- | --- | --- | --- | --- | --- | --- | --- | --- | --- | --- | --- | --- | --- | --- | --- | --- | --- | --- | --- | --- | --- | --- | --- | --- | --- | --- | --- | --- | --- | --- | --- | --- |
|  |  | **TIC** | | | **CFZ** | | | **CEP** | | | **FEP** | | | **CTX** | | | **CRO** | | | **CAZ** | | | **ATM** | | | **ETP** | | | **IMI** | | | **MEM** | | | **CIP** | | | **Lvx** | | | **OFX** | | | **NOR** | | | **TET** | | |
|  |  | S | I | R | S | I | R | S | I | R | S | I | R | S | I | R | S | I | R | S | I | R | S | I | R | S | I | R | S | I | R | S | I | R | S | I | R | S | I | R | S | I | R | S | I | R | S | I | R |
| 2006 | S16 | >20 | 15-19 | <14 | > 18 | 15-17 | <14 | >18 | 15-17 | <14 | > 18 | 15-17 | <14 | >23 | 15-22 | <14 | >21 | 14-20 | <13 | >18 | 15-17 | <14 | >22 | 16-21 | <15 | > 19 | 16-18 | <15 | > 16 | 15-14 | <13 | > 16 | 15-14 | <13 | > 21 | 16-20 | <15 | > 17 | 14-16 | <13 | > 16 | 13-15 | < 12 | > 17 | 13-16 | <12 | > 19 | 15-18 | <14 |
| 2007-2008 | S17 - S18 | >20 | 15-19 | <14 | > 18 | 15-17 | <14 | >18 | 15-17 | <14 | > 18 | 15-17 | <14 | >23 | 15-22 | <14 | >21 | 14-20 | <13 | >18 | 15-17 | <14 | >22 | 16-21 | <15 | > 19 | 16-18 | <15 | > 16 | 15-14 | <13 | > 16 | 15-14 | <13 | > 21 | 16-20 | <15 | > 17 | 14-16 | <13 | > 16 | 13-15 | < 12 | > 17 | 13-16 | <12 | > 15 | 12-14 | <11 |
| 2010 | S20 | >20 | 15-19 | <14 |  |  |  | >18 | 15-17 | <14 | > 18 | 15-17 | <14 | >26 | 23-25 | <22 | >23 | 20-22 | <19 | >21 | 18-20 | <17 | >21 | 18-20 | <17 | > 19 | 16-18 | <15 | > 16 | 15-14 | <13 | > 16 | 15-14 | <13 | > 21 | 16-20 | <15 | > 17 | 14-16 | <13 | > 16 | 13-15 | < 12 | > 17 | 13-16 | <12 | > 15 | 12-14 | <11 |
| 2011 | S21 | >20 | 15-19 | <14 | > 23 | 20-22 | <19 | >18 | 15-17 | <14 | > 18 | 15-17 | <14 | >26 | 23-25 | <22 | >23 | 20-22 | <19 | >21 | 18-20 | <17 | >21 | 18-20 | <17 | > 23 | 20-22 | <19 | > 23 | 20-22 | <19 | > 23 | 20-22 | <19 | > 21 | 16-20 | <15 | > 17 | 14-16 | <13 | > 16 | 13-15 | < 12 | > 17 | 13-16 | <12 | > 15 | 12-14 | <11 |
| 2012 | S22 |  |  |  | > 23 | 20-22 | <19 | >18 | 15-17 | <14 | > 18 | 15-17 | <14 | >26 | 23-25 | <22 | >23 | 20-22 | <19 | >21 | 18-20 | <17 | >21 | 18-20 | <17 | > 22 | 19-21 | <18 | > 23 | 20-22 | <19 | > 23 | 20-22 | <19 | > 31 | 21-30 | <20 |  |  |  | > 16 | 13-15 | < 12 | > 17 | 13-16 | <12 | > 15 | 12-14 | <11 |
| 2013 | S23 | >20 | 15-19 | <14 | > 23 | 20-22 | <19 | >18 | 15-17 | <14 | > 18 | 15-17 | <14 | >26 | 23-25 | <22 | >23 | 20-22 | <19 | >21 | 18-20 | <17 | >21 | 18-20 | <17 | > 22 | 19-21 | <18 | > 23 | 20-22 | <19 | > 23 | 20-22 | <19 | > 31 | 21-30 | <20 |  |  |  |  |  |  | > 17 | 13-16 | <12 | > 15 | 12-14 | <11 |
| 2014 - 2015 | S24 - S25 | >20 | 15-19 | <14 | > 23 | 20-22 | <19 | >18 | 15-17 | <14 | > 25 |  | <18 | >26 | 23-25 | <22 | >23 | 20-22 | <19 | >21 | 18-20 | <17 | >21 | 18-20 | <17 | > 22 | 19-21 | <18 | > 23 | 20-22 | <19 | > 23 | 20-22 | <19 | > 31 | 21-30 | <20 |  |  |  |  |  |  | > 17 | 13-16 | <12 | > 15 | 12-14 | <11 |
| 2016 -2019 | S26 - S29 |  |  |  | > 23 | 20-22 | <19 |  |  |  | > 25 |  | <18 | >26 | 23-25 | <22 | >23 | 20-22 | <19 | >21 | 18-20 | <17 | >21 | 18-20 | <17 | > 22 | 19-21 | <18 | > 23 | 20-22 | <19 | > 23 | 20-22 | <19 | > 31 | 21-30 | <20 |  |  |  |  |  |  |  |  |  | > 15 | 12-14 | <11 |

Green boxes highlight differences between standards

Table S3*.*

Breakpoints comparison among M31 standards by MIC.

Breakpoints comparison among M31 standards by Disk Diffusion.

| **Author** | **Year of the standard publication** | **CLSI Standard** | **CFP**  **mm** | | | **STR**  **mm** | | | **NAL**  **mm** | | | **TMP**  **mm** | | | **TET**  **mm** | | |
| --- | --- | --- | --- | --- | --- | --- | --- | --- | --- | --- | --- | --- | --- | --- | --- | --- | --- |
|  |  |  | S | I | R | S | I | R | S | I | R | S | I | R | S | I | R |
| Van.., et al., (2007) | 2005 | CLSI, M31-A2 |  |  |  | > 15 | 12-14 | <11 | > 19 | 14-18 | < 13 |  |  |  |  |  |  |
| Álvarez-Fernández E, et al., (2012) | 2007 | CLSI, M31-A2 |  |  |  | > 15 | 12-14 | <11 | > 19 | 14-18 | < 13 |  |  |  | > 15 | 12-14 | <11 |
| Zdragas A., et al, (2012) | 2008 | CLSI, M31-A3 |  |  |  |  |  |  |  |  |  |  |  |  | > 19 | 15-18 | <14 |
| Thung TY et al., (2016) | 2008 | CLSI, M31-A3; M31-S1 |  |  |  |  |  |  |  |  |  |  |  |  | > 19 | 15-18 | <14 |

| **Author** | **Year of the standard publication** | **CLSI Standard** | **CTX**  **μg/mL** | | | | **CRO**  **μg/mL** | | | **FOX**  **μg/mL** | | | **STR**  **μg/mL** | | | **CIP**  **μg/mL** | | | **NAL**  **μg/mL** | | |
| --- | --- | --- | --- | --- | --- | --- | --- | --- | --- | --- | --- | --- | --- | --- | --- | --- | --- | --- | --- | --- | --- |
|  |  |  | S | I | R | S | | I | R | S | I | R | S | I | R | S | I | R | S | I | R |
| [4] | 2002 | CLSI, M31-A2 |  |  |  |  | |  |  |  |  |  |  |  |  |  |  |  |  |  |  |
| [10] | 2004 | CLSI, M31-S1 |  |  |  |  | |  |  |  |  |  |  |  |  |  |  |  |  |  |  |
| [9] | 2008 | CLSI, M31-A3 | < 8 | 16-32 | > 64 | < 8 | | 16-32 | > 64 | < 8 | 16 | 32 | < 32 |  | > 64 | ≤ 2 | 4 | > 8 | ≤ 16 |  | > 32 |
| [11] | 2008 | CLSI, M31-A3 | < 8 | 16-32 | > 64 | < 8 | | 16-32 | > 64 | < 8 | 16 | 32 | < 32 |  | > 64 | ≤ 2 | 4 | > 8 | ≤ 16 |  | > 32 |

Blue boxes demonstrate antibiotics where breakpoints were not referenced

Yellow boxes show breakpoints used para la evaluación de betalactamasas.

**References**

1. Aslam M, Checkley S, Avery B, Chalmers G, Bohaychuk V, Gensler G, et al. Phenotypic and genetic characterization of antimicrobial resistance in Salmonella serovars isolated from retail meats in Alberta, Canada. Food Microbiology. 2012;32:110-7; doi: <http://dx.doi.org/10.1016/j.fm.2012.04.017>.

2. Bosilevac JM, Guerini MN, Kalchayanand N, Koohmaraie M. Prevalence and Characterization of Salmonellae in Commercial Ground Beef in the United States. Applied and Envinronmental Microbiology. 2009;75(7):1892–900; doi: <http://dx.doi.org/10.1128/AEM.02530-08>.

3. Fakhr MK, Sherwood JS, Thorsness J, Logue CM. Molecular Characterization and Antibiotic Resistance Profiling of Salmonella Isolated from Retail Turkey Meat Products. Fooborne Pathogens and Disease. 2006;3(4):366-74.

4. MIkanatha NM, Sandt CH, Localio AR, Tewari D, Rankin SC, Whichard JM, et al. Multidrug-Resistant Salmonella Isolates from Retail Chicken Meat Compared with Human Clinical Isolates. Fooborne Pathogens and Disease. 2010;7(8):929-34; doi: <http://dx.doi.org/10.1089=fpd.2009.0499>.

5. Gad AH, Abo-Shama UH, Harclerode KK, Fakhr MK. Prevalence, Serotyping, Molecular Typing, and Antimicrobial Resistance of Salmonella Isolated From Conventional and Organic Retail Ground Poultry. Frontiers in Microbiology. 2018;9:Article 2653; doi: <http://dx.doi.org/10.3389/fmicb.2018.02653>.

6. Donado-Godoy P, Byrne BA, Hume M, Leon M, Perez-Gutierrez E, Vives Flores MJ, et al. Molecular Characterization of *Salmonella* Paratyphi B dT+ and *Salmonella* Heidelberg from Poultry and Retail Chicken Meat in Colombia by Pulsed-Field Gel Electrophoresis. Journal of Food Protection. 2015;78(4):802-7; doi: <http://dx.doi.org/10.4315/0362-028X.JFP-14-356>.

7. Donado-Godoy P, Clavijo V, León M, Arevalo A, Castellanos R, Bernal J, et al. Counts, Serovars, and Antimicrobial Resistance Phenotypes of Salmonella on Raw Chicken Meat at Retail in Colombia. Journal of Food Protection. 2014;77(2):227-35; doi: <http://dx.doi.org/10.4315/0362-028X.JFP-13-276>.

8. Clemente L, Manageiro V, Ferreira E, Jones-Dias D, Correia I, Themudo P, et al. Occurrence of extended-spectrum β-lactamases among isolates of *Salmonella enterica* subsp. enterica from food-producing animals and food products, in Portugal. International Journal of Food Microbiology. 2013;167:221-8; doi: <http://dx.doi.org/10.1016/j.ijfoodmicro.2013.08.009>.

9. Yang B, Cui Y, Shi C, Wang J, Xia X, Xi M, et al. Counts, Serotypes, and Antimicrobial Resistance of Salmonella Isolates on Retail Raw Poultry in the People’s Republic of China. Journal of Food Protection. 2014;77(6):894-902; doi: <http://dx.doi.org/10.4315/0362-028X.JFP-13-439>.

10. Nunes Medeiros MA, Nunes de Oliveira DC, Rodrigues DdP, Coradi de Freitas DR. Prevalence and antimicrobial resistance of Salmonella in chicken carcasses at retail in 15 Brazilian cities. Rev Panam Salud Publica. 2011;30(6):555-60.

11. Tirziu E, Lazar R, Sala C, Nichita I, Morar A, Ere M, et al. Salmonella in Raw Chicken Meat from the Romanian Seaside: Frequency of Isolation and Antibiotic Resistance. Journal o f Food Protection. 2016;78(5):1003-6; doi: <http://dx.doi.org/10.4315/0362-028X.JFP-14-460>.
