# Supplementary material for: Analysis of the assessment of antimicrobial susceptibility. Non-typhoid Salmonella in meat and meat products as model (systematic review)
Source: BMC Microbiol. 2021 Aug 2;21:223. doi: 10.1186/s12866-021-02268-1 (PMC8328484; doi:10.1186/s12866-021-02268-1)
Supplement: Supplementary file 2 — Additional file 2:. A. Selected articles relationship between year of the isolation Vs. Standard used. B. Selected articles relationship between Submission article year Vs. Standard used. C. Selected articles relationship between publication article year Vs. Standard used [16, 17, 19, 23, 27, 31, 34, 37, 39, 41, 44, 46, 47, 52, 56–58, 60, 61, 63, 65, 68–71, 73, 74, 76, 79, 81–88] [file 12866_2021_2268_MOESM2_ESM.docx]

**Table 3. A.** Selected articles relationship between year of the isolation Vs. Standard used

| **Chronological relationship** | | | **Year of standards** | | | | | | | | | | | | | | | | | | | | | **References** |
| --- | --- | --- | --- | --- | --- | --- | --- | --- | --- | --- | --- | --- | --- | --- | --- | --- | --- | --- | --- | --- | --- | --- | --- | --- |
|  |  |  | **1999** | **2000** | **2001** | **2002** | **2003** | **2004** | **2005** | **2006** | **2007** | **2008** | **2009** | **2010** | **2011** | **2012** | **2013** | **2014** | **2015** | **2016** | **2017** | **2018** | **2019** |  |
| **Isolating year** | **Submitting date** | **Publishing date** | **S9** | **S10** | **S11** | **S12** | **S13** | **S14** | **S15** | **S16** | **S17** | **S18** | **S19** | **S20** | **S21** | **S22** | **S23** | **S24** | **S25** | **S26** | **S27** | **S28** | **S29** |  |
| **2002** | Oct-06 | Dec-07 |  |  |  |  |  |  | X |  |  |  |  |  |  |  |  |  |  |  |  |  |  | [27] |
| **2003** | ND | Dec-06 |  |  | X |  |  |  |  |  |  |  |  |  |  |  |  |  |  |  |  |  |  | [23] |
| **2005** | Sep-07 | Mar-09 |  |  |  |  |  |  | X |  |  |  |  |  |  |  |  |  |  |  |  |  |  | [31] |
| **2006** | ND | Nov-10 |  |  |  |  |  |  |  | X |  |  |  |  |  |  |  |  |  |  |  |  |  | [39] |
| **2008** | May-11 | Oct-12 |  |  |  |  |  |  |  |  |  |  |  | X |  |  |  |  |  |  |  |  |  | [44] |
| **2008** | Aug-13 | Jun-14 |  |  |  |  |  |  |  | X |  |  |  |  |  |  |  |  |  |  |  |  |  | [57] |
| **2009** | Jun-10 | Sep-10 |  |  |  |  |  |  |  |  |  |  | X |  |  |  |  |  |  |  |  |  |  | [37] |
| **2009** | Aug-10 | Jan-11 | X |  |  |  |  |  |  |  |  |  |  |  |  |  |  |  |  |  |  |  |  | [41] |
| **2009** | ND | Nov-12 |  |  |  |  |  |  |  |  |  |  |  | X |  |  |  |  |  |  |  |  |  | [46] |
| **2009** | ND | Oct-14 |  |  |  |  |  |  |  |  | X |  |  |  |  |  |  |  |  |  |  |  |  | [61] |
| **2009** | Jul-14 | Apr-15 |  |  |  |  |  |  |  |  |  |  |  |  |  | X |  |  |  |  |  |  |  | [70] |
| **2009** | Jun-18 | 2018 |  |  |  |  |  |  |  |  |  |  |  |  | X |  |  |  |  |  |  |  |  | [82] |
| **2010** | Mar-13 | Dec-13 |  |  |  |  |  |  |  |  |  | X |  |  |  |  |  |  |  |  |  |  |  | [52] |
| **2011** | ND | Sep-12 |  |  |  |  |  |  |  |  |  |  |  |  | X |  |  |  |  |  |  |  |  | [47] |
| **2012** | Jun-13 | Feb-14 |  |  |  |  |  |  |  |  |  |  |  |  |  | X |  |  |  |  |  |  |  | [56] |
| **2012** | Jul-13 | Feb-14 |  |  |  |  |  |  |  |  |  |  |  |  |  |  | X |  |  |  |  |  |  | [63] |
| **2012** | Mar-14 | Jul-14 |  |  | X |  |  |  |  |  |  |  |  |  |  |  |  |  |  |  |  |  |  | [16] |
| **2013** | Jan-14 | Sep-14 |  |  |  |  |  |  |  |  |  |  |  |  |  | X |  |  |  |  |  |  |  | [58] |
| **2014** | Sep-14 | May-15 |  |  |  |  |  |  |  | X |  |  |  |  |  |  |  |  |  |  |  |  |  | [68] |
| **2014** | Apr-15 | Nov-15 |  |  |  |  |  |  |  |  |  |  |  |  |  |  | X |  |  |  |  |  |  | [69] |
| **2014** | May-15 | Apr-16 |  |  |  |  |  |  |  |  |  |  |  |  |  |  | X |  |  |  |  |  |  | [73] |
| **2014** | Jul-15 | Jan-16 |  |  |  |  |  |  |  |  |  |  |  |  |  |  |  | X |  |  |  |  |  | [74] |
| **2014** | Jul-18 | 2018 |  |  |  |  |  |  |  |  |  |  |  |  |  |  | X |  |  |  |  |  |  | [85] |
| **2015** | Sep-15 | Dec-15 |  |  |  |  |  |  |  |  |  |  |  | X |  |  |  |  |  |  |  |  |  | [71] |
| **2015** | ND | Jan-16 |  |  |  |  |  |  |  |  |  |  |  |  |  |  | X |  |  |  |  |  |  | [76] |
| **2015** | Feb-16 | Nov-16 |  |  |  |  |  |  |  |  |  |  |  |  |  |  | X |  |  |  |  |  |  | [19] |

1. Selected articles relationship between Submission article year V*s.* Standard used

| **Chronological relationship** | | | **Year of standards** | | | | | | | | | | | | | | | | | | | | | **References** |
| --- | --- | --- | --- | --- | --- | --- | --- | --- | --- | --- | --- | --- | --- | --- | --- | --- | --- | --- | --- | --- | --- | --- | --- | --- |
|  |  |  | **1999** | **2000** | **2001** | **2002** | **2003** | **2004** | **2005** | **2006** | **2007** | **2008** | **2009** | **2010** | **2011** | **2012** | **2013** | **2014** | **2015** | **2016** | **2017** | **2018** | **2019** |  |
| **Submitting date** | **Isolating year** | **Publishing date** | **S9** | **S10** | **S11** | **S12** | **S13** | **S14** | **S15** | **S16** | **S17** | **S18** | **S19** | **S20** | **S21** | **S22** | **S23** | **S24** | **S25** | **S26** | **S27** | **S28** | **S29** |  |
| **Oct-06** | 2002 | Dec-07 |  |  |  |  |  |  | X |  |  |  |  |  |  |  |  |  |  |  |  |  |  | [27] |
| **Sep-07** | 2005 | Mar-09 |  |  |  |  |  |  | X |  |  |  |  |  |  |  |  |  |  |  |  |  |  | [31] |
| **Jan-09** | 2006-2007 | 2010 |  |  |  |  | X |  |  | X |  |  |  |  |  |  |  |  |  |  |  |  |  | [34] |
| **Jun-10** | 2009 | Sep-10 |  |  |  |  |  |  |  |  |  |  | X |  |  |  |  |  |  |  |  |  |  | [37] |
| **Aug-10** | 2009 | Jan-11 | X |  |  |  |  |  |  |  |  |  |  |  |  |  |  |  |  |  |  |  |  | [41] |
| **May-11** | 2008 | Oct-12 |  |  |  |  |  |  |  |  |  |  |  | X |  |  |  |  |  |  |  |  |  | [44] |
| **Mar-13** | 2010 | Dec-13 |  |  |  |  |  |  |  |  |  | X |  |  |  |  |  |  |  |  |  |  |  | [52] |
| **Mar-13** | ND | 2014 |  |  |  |  |  |  |  |  |  |  |  |  |  | X |  |  |  |  |  |  |  | [17] |
| **May-13** | ND | 2014 |  |  |  |  |  |  |  |  |  |  |  |  | X |  |  |  |  |  |  |  |  | [65] |
| **Jun-13** | 2012 | Feb-14 |  |  |  |  |  |  |  |  |  |  |  |  |  | X |  |  |  |  |  |  |  | [56] |
| **Jul-13** | 2012 | Feb -14 |  |  |  |  |  |  |  |  |  |  |  |  |  |  | X |  |  |  |  |  |  | [63] |
| **Aug-13** | 2008 | Jun-14 |  |  |  |  |  |  |  | X |  |  |  |  |  |  |  |  |  |  |  |  |  | [57] |
| **Jan-14** | 2013 | Sep -14 |  |  |  |  |  |  |  |  |  |  |  |  |  | X |  |  |  |  |  |  |  | [58] |
| **Feb-14** | ND | 2014 |  |  |  |  |  |  |  |  |  |  |  |  | X |  |  |  |  |  |  |  |  | [60] |
| **Mar-14** | 2012 | Jul -14 |  |  | X |  |  |  |  |  |  |  |  |  |  |  |  |  |  |  |  |  |  | [16] |
| **Jul-14** | 2009 | Apr-15 |  |  |  |  |  |  |  |  |  |  |  |  |  | X |  |  |  |  |  |  |  | [70] |
| **Sep-14** | 2014 | May -15 |  |  |  |  |  |  |  | X |  |  |  |  |  |  |  |  |  |  |  |  |  | [68] |
| **Apr-15** | 2014 | Nov-15 |  |  |  |  |  |  |  |  |  |  |  |  |  |  | X |  |  |  |  |  |  | [69] |
| **May-15** | 2014 | Apr-16 |  |  |  |  |  |  |  |  |  |  |  |  |  |  | X |  |  |  |  |  |  | [73] |
| **Jul-15** | 2014 | Jan-16 |  |  |  |  |  |  |  |  |  |  |  |  |  |  |  | X |  |  |  |  |  | [74] |
| **Sep-15** | 2015 | Dec-15 |  |  |  |  |  |  |  |  |  |  |  | X |  |  |  |  |  |  |  |  |  | [71] |
| **Feb-16** | 2015 | Nov-16 |  |  |  |  |  |  |  |  |  |  |  |  |  |  | X |  |  |  |  |  |  | [19] |
| **Apr-17** | ND | 2017 |  |  |  |  |  |  |  |  |  |  |  |  |  |  |  |  | X |  |  |  |  | [79] |
| **Jan-18** | 2016-2017 | 2018 |  |  |  |  |  |  |  |  |  |  |  |  |  |  | X |  |  |  |  |  |  | [83] |
| **Jan-18** | 2015-2016 | 2018 |  |  |  |  |  |  |  |  |  |  |  |  |  |  |  | X |  |  |  |  |  | [86] |
| **Jun-18** | 2009 | 2018 |  |  |  |  |  |  |  |  |  |  |  |  | X |  |  |  |  |  |  |  |  | [82] |
| **Jul-18** | 2014 | 2018 |  |  |  |  |  |  |  |  |  |  |  |  |  | X |  |  |  |  |  |  |  | [85] |
| **Apr-18** | 2016-2017 | 2019 |  |  |  |  |  |  |  |  |  |  |  |  |  |  | X |  |  |  |  |  |  | [87] |
| **Apr -19** | 2011-2016 | 2019 |  |  |  |  |  |  |  |  |  |  |  |  |  |  |  |  |  |  |  | X |  | [88] |

1. Selected articles relationship between publication article year Vs. Standard used

| **Chronological relationship** | | | **Year of standards** | | | | | | | | | | | | | | | | | | | | |  |
| --- | --- | --- | --- | --- | --- | --- | --- | --- | --- | --- | --- | --- | --- | --- | --- | --- | --- | --- | --- | --- | --- | --- | --- | --- |
|  |  |  | **1999** | **2000** | **2001** | **2002** | **2003** | **2004** | **2005** | **2006** | **2007** | **2008** | **2009** | **2010** | **2011** | **2012** | **2013** | **2014** | **2015** | **2016** | **2017** | **2018** | **2019** | **References** |
| **Publishing date** | **Submitting date** | **Isolating year** | **S9** | **S10** | **S11** | **S12** | **S13** | **S14** | **S15** | **S16** | **S17** | **S18** | **S19** | **S20** | **S21** | **S22** | **S23** | **S24** | **S25** | **S26** | **S27** | **S28** | **S29** |  |
| **Dec-06** | ND | 2003 |  |  | X |  |  |  |  |  |  |  |  |  |  |  |  |  |  |  |  |  |  | [23] |
| **Dec-07** | Oct-06 | 2002 |  |  |  |  |  |  | X |  |  |  |  |  |  |  |  |  |  |  |  |  |  | [27] |
| **Mar-09** | Sep -07 | 2005 |  |  |  |  |  |  | X |  |  |  |  |  |  |  |  |  |  |  |  |  |  | [31] |
| **2010** | Jan-09 | 2006-2007 |  |  |  |  | X |  |  | X |  |  |  |  |  |  |  |  |  |  |  |  |  | [34] |
| **Nov-10** | ND | 2006 |  |  |  |  |  |  |  | X |  |  |  |  |  |  |  |  |  |  |  |  |  | [39] |
| **Sep-10** | Jun-10 | 2009 |  |  |  |  |  |  |  |  |  |  | X |  |  |  |  |  |  |  |  |  |  | [37] |
| **Jan-11** | Aug-10 | 2009 | X |  |  |  |  |  |  |  |  |  |  |  |  |  |  |  |  |  |  |  |  | [41] |
| **Oct-12** | May-11 | 2008 |  |  |  |  |  |  |  |  |  |  |  | X |  |  |  |  |  |  |  |  |  | [44] |
| **Nov-12** | ND | 2009 |  |  |  |  |  |  |  |  |  |  |  | X |  |  |  |  |  |  |  |  |  | [46] |
| **Sep-12** | ND | 2011 |  |  |  |  |  |  |  |  |  |  |  |  | X |  |  |  |  |  |  |  |  | [47] |
| **Dec-13** | Mar-13 | 2010 |  |  |  |  |  |  |  |  |  | X |  |  |  |  |  |  |  |  |  |  |  | [52] |
| **Jun-14** | Aug-13 | 2008 |  |  |  |  |  |  |  | X |  |  |  |  |  |  |  |  |  |  |  |  |  | [57] |
| **Oct-14** | ND | 2009 |  |  |  |  |  |  |  |  | X |  |  |  |  |  |  |  |  |  |  |  |  | [61] |
| **Feb-14** | Jun-13 | 2012 |  |  |  |  |  |  |  |  |  |  |  |  |  | X |  |  |  |  |  |  |  | [56] |
| **2014** | Feb-14 | ND |  |  |  |  |  |  |  |  |  |  |  |  | X |  |  |  |  |  |  |  |  | [60] |
| **2014** | May-13 | ND |  |  |  |  |  |  |  |  |  |  |  |  | X |  |  |  |  |  |  |  |  | [65] |
| **Sep-14** | Jan-14 | 2013 |  |  |  |  |  |  |  |  |  |  |  |  |  | X |  |  |  |  |  |  |  | [58] |
| **2014** | Mar-13 | ND |  |  |  |  |  |  |  |  |  |  |  |  |  | X |  |  |  |  |  |  |  | [17] |
| **Feb-14** | Jul-13 | 2012 |  |  |  |  |  |  |  |  |  |  |  |  |  |  | X |  |  |  |  |  |  | [63] |
| **Jul-14** | Mar-14 | 2012 |  |  | X |  |  |  |  |  |  |  |  |  |  |  |  |  |  |  |  |  |  | [16] |
| **May-15** | Sep-14 | 2014 |  |  |  |  |  |  |  | X |  |  |  |  |  |  |  |  |  |  |  |  |  | [68] |
| **Dec-15** | Sep-15 | 2015 |  |  |  |  |  |  |  |  |  |  |  | X |  |  |  |  |  |  |  |  |  | [71] |
| **Apr-15** | Jul-14 | 2009 |  |  |  |  |  |  |  |  |  |  |  |  |  | X |  |  |  |  |  |  |  | [70] |
| **Nov-15** | Apr-15 | 2014 |  |  |  |  |  |  |  |  |  |  |  |  |  |  | X |  |  |  |  |  |  | [69] |
| **Jan-16** | ND | 2015 |  |  |  |  |  |  |  |  |  |  |  |  |  |  | X |  |  |  |  |  |  | [76] |
| **Nov-16** | Feb-16 | 2015 |  |  |  |  |  |  |  |  |  |  |  |  |  |  | X |  |  |  |  |  |  | [19] |
| **Apr-16** | May-15 | 2014 |  |  |  |  |  |  |  |  |  |  |  |  |  |  | X |  |  |  |  |  |  | [73] |
| **Jan-16** | Jul-15 | 2014 |  |  |  |  |  |  |  |  |  |  |  |  |  |  |  | X |  |  |  |  |  | [74] |
| **2017** | ND | ND |  |  |  |  |  |  |  |  |  |  |  |  | X |  |  |  |  |  |  |  |  | [81] |
| **2017** | Apr-17 | ND |  |  |  |  |  |  |  |  |  |  |  |  |  |  |  |  | X |  |  |  |  | [79] |
| **2018** | Jun-18 | 2009 |  |  |  |  |  |  |  |  |  |  |  |  | X |  |  |  |  |  |  |  |  | [82] |
| **2018** | Jan-18 | 2016-2017 |  |  |  |  |  |  |  |  |  |  |  |  |  |  | X |  |  |  |  |  |  | [83] |
| **2018** | ND | 2016-2017 |  |  |  |  |  |  |  |  |  |  |  |  |  |  |  | X |  |  |  |  |  | [84] |
| **2018** | Jul-18 | 2014 |  |  |  |  |  |  |  |  |  |  |  |  |  | X |  |  |  |  |  |  |  | [85] |
| **2018** | Jan-18 | 2015-2016 |  |  |  |  |  |  |  |  |  |  |  |  |  |  |  | X |  |  |  |  |  | [23] |
| **2019** | 2018 | 2016-2017 |  |  |  |  |  |  |  |  |  |  |  |  |  |  | X |  |  |  |  |  |  | [27] |
| **2019** | 2019 | 2011-2016 |  |  |  |  |  |  |  |  |  |  |  |  |  |  |  |  |  |  |  | X |  | [31] |

In Tables 3 A, B, and C. X indicates the year and the standard implemented in the study, in red letters the articles in which there is an impact on the modification of the breakpoints, in yellow the articles that used norms outside the range considered optimal period, the green boxes the optional period standards for the analysis of the results based on the date of isolation, submission, and publication, the blue boxes indicate the related standard according to the year of isolation, submission, or publication of the article.
